# Supplementary material for: Testing CPT symmetry in ortho-positronium decays with positronium annihilation tomography
Source: Nat Commun. 2021 Sep 27;12:5658. doi: 10.1038/s41467-021-25905-9 (PMC8476595; doi:10.1038/s41467-021-25905-9)
Supplement: Supplementary file 1 — Supplementary Information [file 41467_2021_25905_MOESM1_ESM.pdf]

Supplementary Information for

Testing CPT symmetry in ortho-positronium decays with positronium annihilation tomography

P. Moskal[1,2,*], A. Gajos[1,2,**], M. Mohammed[1], J. Chhokar[1,2], N. Chug[1,2], C. Curceanu[3], E. Czerwiński[1,2], M. Dadgar[1,2], K. Dulski[1,2], M. Gorgol[4], J. Goworek[5], B. C. Hiesmayr[6], B. Jasińska[4], K. Kacprzak[1], Ł. Kapłon[1,2], H. Karimi[1,2], D. Kisielewska[1], K. Klimaszewski[7], G. Korcyl[1,2], P. Kowalski[7], N. Krawczyk[1,2], W. Krzemień[8], T. Kozik[1], E. Kubicz[1,2], S. Niedźwiecki[1,2], S. Parzych[1,2], M. Pawlik-Niedźwiecka[1,2], L. Raczyński[7], J. Raj[1,2], S. Sharma[1,2], Shivani[1,2], R.Y. Shopa[7], A. Sienkiewicz[5], M. Silarski[1,2], M. Skurzok[1,3], E. Ł. Stępień[1,2], F. Tayefi[1,2], and W. Wiślicki[7]

[1]Faculty of Physics, Astronomy and Applied Computer Science, Jagiellonian University, S. Łojasiewicza 11, 30-348 Kraków, Poland
[2]Total-Body Jagiellonian-PET Laboratory, Jagiellonian University, Poland
[3]INFN, Laboratori Nazionali di Frascati CP 13, Via E. Fermi 40, 00044, Frascati, Italy
[4]Department of Nuclear Methods, Institute of Physics, Maria Curie-Skłodowska University, Pl. M. Curie-Skłodowskiej 1, 20-031 Lublin, Poland
[5]Faculty of Chemistry, Institute of Chemical Sciences, Maria Curie-Skłodowska University, Pl. M. Curie-Skłodowskiej 3, 20-031 Lublin, Poland
[6]Faculty of Physics, University of Vienna Boltzmanngasse 5, 1090 Vienna, Austria
[7]Department of Complex Systems, National Centre for Nuclear Research, 05-400 Otwock-Świerk, Poland
[8]High Energy Department, National Centre for Nuclear Research, 05-400 Otwock-Świerk, Poland

*corresponding author, e-mail: p.moskal@uj.edu.pl
**corresponding author, e-mail: aleksander.gajos@uj.edu.pl

Supplementary Note 1: Study of the sensitivity of the experimental method to CPT-violating effects

In order to demonstrate the sensitivity of the experimental approach to potential CPT-violating effects in the cosθ operator distribution, a set of Monte Carlo simulation experiments was performed in which different levels of a CPT-violating asymmetry was introduced into the distribution of the main observable, the cosθ operator.

The simulations included the allowed angular and energy distributions of photons from o-Ps→3γ annihilations expected from quantum electrodynamics, materials and geometry of the positronium production setup as well as of the detector, Compton interactions of the annihilation photons and photon registration threshold on deposited energy characteristic of the detection setup. Realistic resolutions of the J-PET detector were included in the recorded interaction times and deposited energies as well as in their spatial location. Each simulation was performed with the same statistics as that of the experimental data, resulting in a comparable statistical sensitivity. Simulated events were subjected to the same selection and analysis criteria as data. In order to minimize the impact of statistical fluctuations, multiple independent simulations were performed for each value of the induced asymmetry in the 10^{-5}-10^{-2} range.

Supplementary Figure 1 presents the obtained dependence of the values of the C_{CPT} coefficient extracted from the analysed simulations on the CPT asymmetry levels assumed at the MC generation stage. It shows that the presented approach with the current statistics of the experimental dataset is sensitive to CPT-violating asymmetries in the distribution of the cosθ operator with magnitudes starting at about 5×10^{-4} whereas for smaller asymmetries the obtained values are consistently null results, showing no false asymmetries originating from the detector or data analysis.

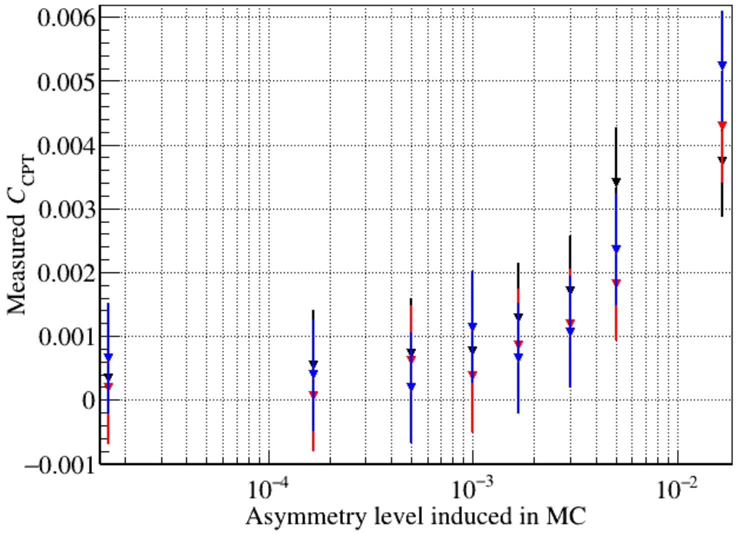

Supplementary Figure 1: Sensitivity of the experiment to CPT-violating asymmetries induced in Monte Carlo simulations. Values of C_{CPT} coefficient obtained from analysis of MC simulations as a function of the level of CPT asymmetry induced in the simulations. Analysis was performed at the same way as the analysis of the experimental data also including the experimental analysing power of 0.37. For each value of assumed asymmetry, different colours denote independent simulations of the same case of CPT violation. The error bars denote statistical uncertainty. The analysis applied to simulated events was identical as used on experimental data and the statistics of simulations was matching that of data.
